# SGLT2-inhibitors; more than just glycosuria and diuresis

**DOI:** 10.1007/s10741-020-10038-w

**Published:** 2020-12-04

**Authors:** Amir Fathi, Keeran Vickneson, Jagdeep S. Singh

**Affiliations:** 1 Department of Neuroanaesthesia and Critical Care, National Hospital for Neurology and Neurosurgery, University College London, London, UK; 2 Division of Molecular and Clinical Medicine, School of Medicine, University of Dundee, Dundee, UK; 3 Department of Cardiology, The Edinburgh Heart Center, Royal Infirmary of Edinburgh, 51 Little France Crescent, Edinburgh, EH16 4SA UK

**Keywords:** SGLT2-inhibitors, Renal disease, Heart failure, Calcium handling, Myocardial energetics, Ventricular remodelling

## Abstract

Heart failure (HF) continues to be a serious public health challenge despite significant advancements in therapeutics and is often complicated by multiple other comorbidities. Of particular concern is type 2 diabetes mellitus (T2DM) which not only amplifies the risk, but also limits the treatment options available to patients. The sodium-glucose linked cotransporter subtype 2 (SGLT2)-inhibitor class, which was initially developed as a treatment for T2DM, has shown great promise in reducing cardiovascular risk, particularly around HF outcomes – regardless of diabetes status.

There are ongoing efforts to elucidate the true mechanism of action of this novel drug class. Its primary mechanism of inducing glycosuria and diuresis from receptor blockade in the renal nephron seems unlikely to be responsible for the rapid and striking benefits seen in clinical trials. Early mechanistic work around conventional therapeutic targets seem to be inconclusive. There are some emerging theories around its effect on myocardial energetics and calcium balance as well as on renal physiology. In this review, we discuss some of the cutting-edge hypotheses and concepts currently being explored around this drug class in an attempt better understand the molecular mechanics of this novel agent.

## Introduction

Heart failure (HF) is a growing public health concern with 8.5 million people expected to be living with this condition in the year 2030 at a staggering cost of $70 billion in the United States alone [1]. HF not only shortens life, but also reduces its quality. There have been considerable advancements in the management of this disease using a variety of neurohormonal modulators and, more recently, with device therapy. Nevertheless, HF remains a challenge to treat particularly because it is frequently associated with other co-morbidities such as hypertension, type 2 diabetes mellitus (T2DM), ischaemic heart disease and renal impairment. Ischaemic heart disease is the commonest cause of heart failure and T2DM amplifies that risk further. Patients with ischaemic cardiomyopathy and T2DM face a doubling of mortality risk compared to patients without T2DM. Indeed, T2DM increases mortality risk regardless of the underlying aetiology of heart failure [2].

Interestingly, there appears to be a bidirectional relationship between T2DM and HF; not only can T2DM cause HF, but it can also be the consequence of it [3]. This underscores the importance of optimal diabetes control in patients with HF. Although there is a multitude of efficacious medications for T2DM, they do not appear to reduce the risk of cardiovascular (CV) outcomes and in the context of HF—some of these agents may even be harmful. A meta-analysis involving 95,000 individuals showed a 42% increased risk of incident HF with the use of thiazolidinediones in patients with T2DM while DPPIV-inhibitors increased that risk by 25% [4]. Another meta-analysis of large HF trials showed patients with HF using insulin had a 27% increased risk of all-cause mortality [5]. These data highlight the complexity, and potential dangers, of managing T2DM and HF concomitantly.

A novel class of anti-diabetes therapy known as the sodium glucose-linked cotransporter subtype 2 (SGLT2) inhibitor has shown potential in addressing this area of urgent unmet need, not only by improving glycaemia but also preserving renal function and reducing hard HF outcomes as well. In this review, we discuss the evidence behind the striking CV benefits and unpick some of the unique characteristics of this drug class that could potentially herald a new generation of targeted agents in the treatment of HF.

### SGLT2 inhibitors – what it says on the tin

**Fig. 1:**
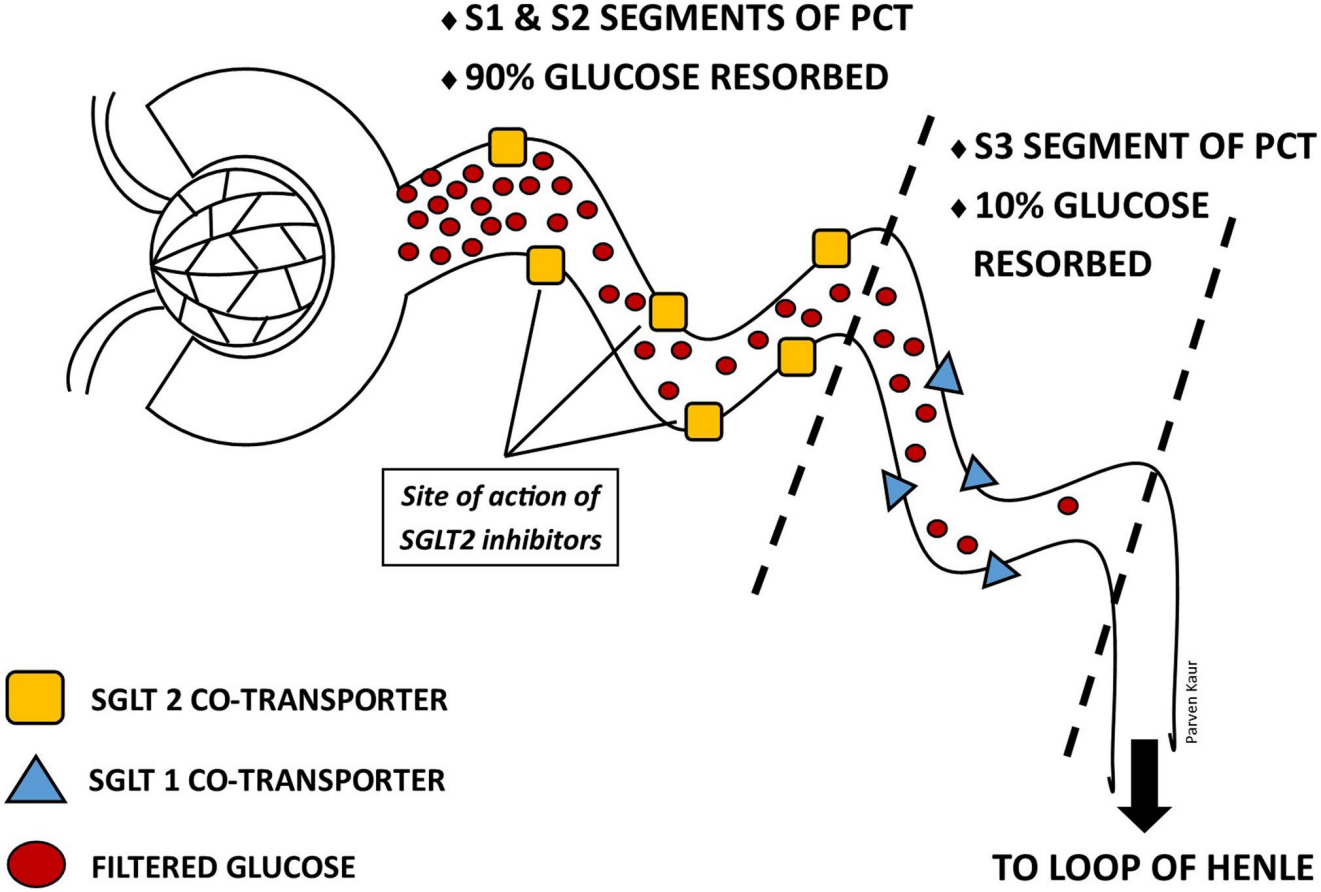
Normal renal tubular resorption of glucose. The diagram also identifies the site at which SGLT2-inhibitors act [8]. Abbreviations: PCT-proximal convoluted tubules; SGLT-sodium-glucose cotransporter

Canagliflozin, dapagliflozin, empagliflozin and ertugliflozin are four currently available agents of the SGLT2-inhibitor class. All four are indicated for T2DM, while dapagliflozin recently received FDA approval for HF, with or without T2DM [6]. As the name implies, SGLT2-inhibitors work by inhibiting the sodium-glucose cotransporter subtype-2, located in the S1 and S2 segments of the proximal convoluted tubule (PCT) of the kidney. SGLT2 is a low affinity high capacity transporter (K_m_ 2 mM) responsible for approximately 90% reabsorption of filtered plasma glucose, and the remainder is reabsorbed by SGLT1 [7]. Potent SGLT2-inhibition prevents the reabsorption of filtered glucose as well as sodium, resulting in glucosuria and natriuresis. (Fig. 1) Other pleotropic benefits of SGLT2-inhibition include weight loss (1.8 to 2.7 kg), reductions in blood pressure (systolic blood pressure (BP): 1.0–2.6 mmHg; diastolic BP 0.7–2.2 mmHg) [9], without increases in heart rate and a low potential of inducing hypoglycaemia [10].

**Fig. 2:**
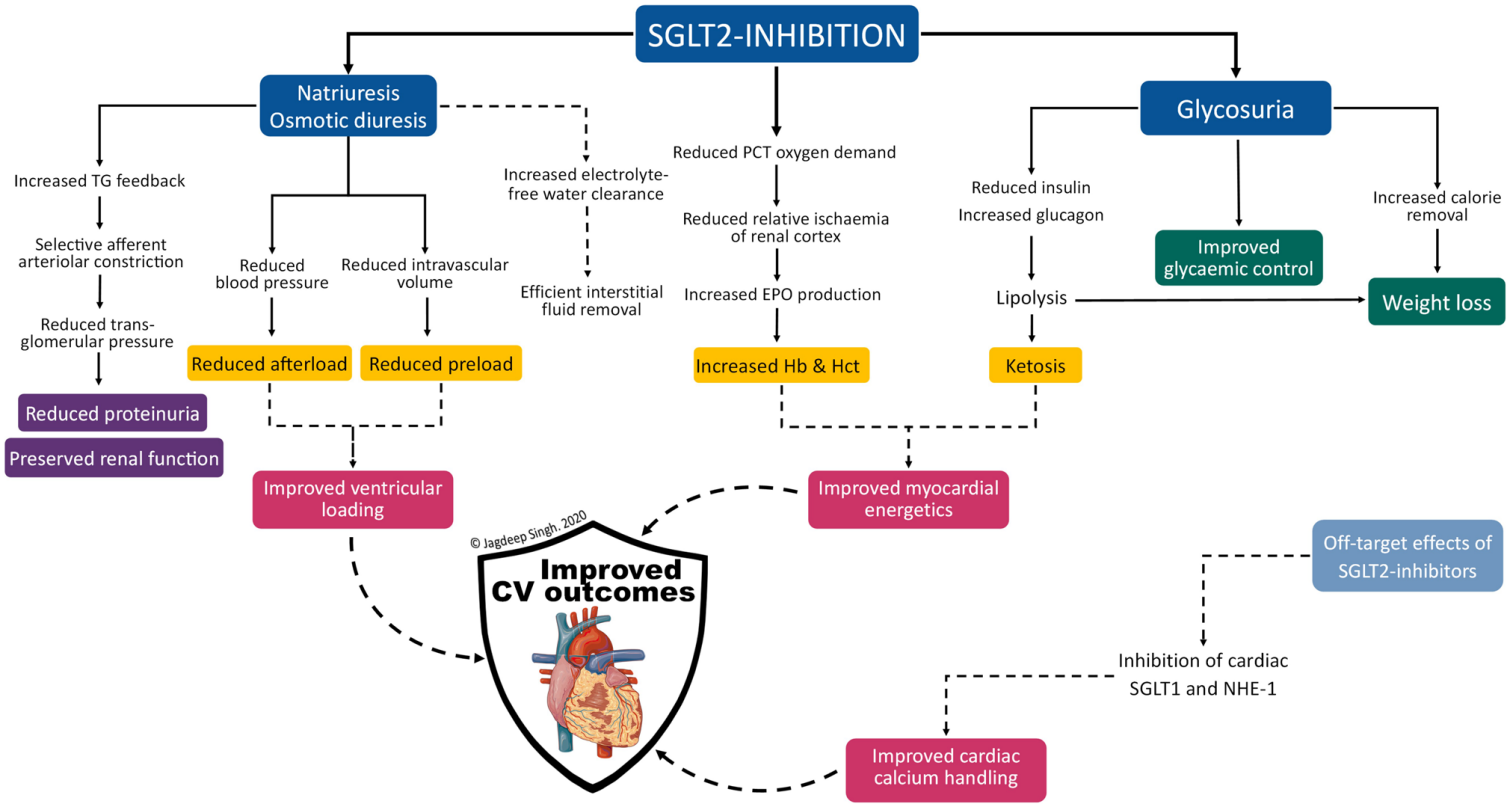
Overview of the effects of SGLT2-inhibitor therapy. Abbreviations: CV-cardiovascular; EPO-erythropoietin; Hb-haemoglobin; Hct-haematocrit; NHE-1- sodium-hydrogen exchanger subtype-1; PCT-proximal convoluted tubule (of renal nephron); SGLT-sodium-glucose linked cotransporter; TG-tubuloglomerular

Unlike other antidiabetic agents, the glucose lowering effect of SGLT2 inhibitors is independent of pancreatic beta-cell function and insulin sensitivity – an ideal property in the context of T2DM disease progression [11] and metabolic milieu of the T2DM phenotype [12]. This insulin-independent fall in plasma glucose reduces insulin requirements and induces a rise in the glucagon-to-insulin ratio, shifting metabolism towards a catabolic state [13]. The caloric loss coupled with increased lipolysis (from catabolism) are responsible for the sustained steady-state weight loss seen with SGLT2-inhibition. (Fig. 2) Body composition studies have shown approximately two-thirds of sustained weight loss is attributable to loss of body fat [14].

In addition, glucosuria and natriuresis simulate an osmotic diuretic effect resulting in reduced plasma volume. Filtered glucose that is not reabsorbed increases tubular fluid osmolarity, flattening the osmotic gradient between tubular fluid and the interstitium thereby reducing electrolyte-free water reabsorption. This is expected to provide some relief on cardiac filling volume and blood pressure which in turn results in reduced preload and afterload respectively. (Fig. 2) Unlike conventional sodium-driven diuretics, SGLT2-inhibititor-mediated free water excretion is expected to be more efficient in relieving signs and symptoms of interstitial congestion without adversely compromising intravascular fluid status or causing reflex sympathetic activation [15, 16]. The recently published RECEDE-CHF trial confirmed that in a cohort of patients with established HF on long-term diuretic therapy, the addition of empagliflozin increased urine output by over 500 ml/day and more than half of that volume was in the form of electrolyte-free water clearance [17].

These are the primary mechanisms of action of the SGLT2-inhibitor class that have been purported to be responsible for its beneficial effects, however as our understanding of this drug class evolves there is a recognition that there may be other novel pathways at play – we explore these pathways in the later sections of this article.

### Cardiovascular outcome trials – the breakthrough

In 2015, the Empagliflozin Cardiovascular Outcome Event Trial in Type 2 Diabetes Mellitus Patients (EMPA-REG OUTCOME) was the first FDA-mandated cardiovascular outcome trial (CVOT) of the class. It studied 7020 individuals with T2DM, randomised to empagliflozin or placebo over a mean follow-up period of 3.1 years [18]. There were striking reductions in 3-point major adverse cardiovascular events (MACE); (HR 0.86; 95% CI 0.74–0.99), all-cause mortality (HR 0.68; 95% CI 0.57–0.82), CV mortality (HR 0.62; 95% CI 0.49–0.77) and HF hospitalisation (HR 0.65; 95% CI 0.50–0.85) [18]. Further analyses revealed the CV benefit with empagliflozin was independent of CV comorbidity burden [19, 20] and renal function [21]. Although the most promising effects of the drug were around HF outcomes, only 10% of the cohort had a diagnosis of HF prior to randomisation and, importantly, there was no formal confirmation / characterisation of HF (e.g. by measuring natriuretic peptides or performing echocardiography). Another unexpected finding was the rapidity of benefit, with the HF hospitalisation and CV mortality curves diverging within weeks of initiation of therapy.

The Canagliflozin Cardiovascular Assessment Study (CANVAS) and Dapagliflozin Effect on Cardiovascular Events-Thrombolysis in Myocardial Infarction 58 (DECLARE-TIMI 58) trials were the next two CVOTs to establish CV safety and efficacy of canagliflozin and dapagliflozin respectively. Both studies utilised a broader inclusion criteria of patients, which included those at high risk of developing CV disease as well as patients with already established CV disease. The majority of participants in CANVAS had established CV disease, while the reverse was true for DECLARE-TIMI 58. Both had a similar proportion of patients with a history of HF to EMPA-REG OUTCOME. Canagliflozin significantly reduced 3-point MACE (HR 0.86; 95% CI 0.75–0.97) and HF hospitalisation (HR 0.67; 95% CI 0.52–0.87), but there was no significant reduction in mortality [22]. Dapagliflozin had no effect on 3-point MACE (HR 0.93; 95% CI 0.84—1.03) or CV death (HR 0.98; 95% CI 0.82–1.17) but there was still significantly lower hospitalisations for HF (HR 0.73; 95% CI 0.61–0.88) [23]. In post-hoc analysis of DECLARE-TIMI 58 where participants were stratified by ejection fraction, dapagliflozin reduced risk of CV death and HF hospitalisation to a greater extent in patients with reduced ejection fraction (EF) [24], however these findings are to be interpreted with caution given the small proportion of patients and lack of robust characterisation of HF. A meta-analysis of all three CVOTs showed an overall 14% reduction in MACE, 23% reduction in composite HF hospitalisation and CV death and 45% reduction in renal disease progression [25]. These findings have also been reproduced in large, real-world clinical studies [26, 27].

Cardiovascular Outcomes Following Ertugliflozin Treatment in Type 2 Diabetes Mellitus Participants With Vascular Disease (VERTIS CV) is the most recent CVOT to investigate effects of ertugliflozin in over 8000 patients with established CV disease [28]. The inclusion criteria used was very similar to EMPA-REG OUTCOME, with a larger proportion of patients with HF at baseline in VERTIS CV (23.1% vs 10.1%). Ertugliflozin failed to demonstrate superiority in the primary outcome of 3-point MACE (HR 0.97 95% CI 0.85–1.11) or any of the key secondary outcomes; CV death (HR 0.92 95% CI 0.77–1.11) and renal disease progression (HR 0.81 95% CI 0.63—1.04). However, a 30% reduction in risk of hospitalisation for HF was seen with ertugliflozin, consistent with the class effect seen in previous CVOTs. The discordant results in CV death or renal disease progression, especially in a secondary prevention cohort is surprising given the benefits seen in other CVOTs; further analysis is awaited.

Findings from these trails led the leading cardiac (and diabetes) societies to recommend the use of SGLT2-inhibitors in patients with coronary artery disease with T2DM to reduce the risk of future CV events [29, 30].

### HF with reduced ejection fraction – SGLT2-inhibitors are fast becoming a must have drug

The Dapagliflozin and Prevention of Adverse outcomes in Heart Failure (DAPA-HF) trial was the first to prospectively investigate the benefits of dapagliflozin in a HF population with reduced ejection fraction (HFrEF) with or without diabetes. Dapagliflozin demonstrated a remarkable reduction in composite CV death or worsening HF (defined as hospitalisation or urgent visit for HF) (HR 0.74 [95% CI 0.65–0.85]), with each component being reduced by 18% and 30% respectively [31]. Patients also experienced less symptoms of HF in the dapagliflozin arm compared to conventional HF therapy as evidenced by a significant and clinically meaningful improvement in Kansas City Cardiomyopathy Questionnaire score. Some noteworthy aspects of the study include: 1) majority of the participants were on well-established background HF therapy (ACEi/ARB/ARNI 94%, BB 96%, MRA 71%), which further underscores the added, incremental benefit of dapagliflozin on CV outcomes; 2) CV benefit manifested early and was maintained throughout course of the study; 3) short median follow-up period of 18 months as compared to other key HF therapies (with the exception of beta-blockers) and 4) pre-specified analyses showed CV benefit was consistent, regardless of diabetes status at baseline and there were no safety concerns regarding hypoglycaemia or adverse volume depletion in non-diabetic HF patients [31].

Empagliflozin Outcome Trial in Patients with Chronic Heart Failure with Reduced Ejection Fraction (EMPEROR-Reduced) was designed to be more ambitious than DAPA-HF, recruiting a HF population that had on average more severe systolic dysfunction (mean EF 27% vs. 31%; N-terminal pro B-type natriuretic peptide (NT-proBNP) 1907 vs. 1437) [32]. After a median follow-up of 16 months, the incidence of primary outcome of CV death or hospitalisation for HF was significantly reduced by empagliflozin therapy (HR 0.75; 95% CI 0.65–0.86]), largely driven by lower HF hospitalisations. [32]. The lack of signal in CV mortality (HR 0.92; 95% CI 0.75–1.12) was somewhat surprising. Perhaps, in patients with more advanced HF, the ability of empagliflozin to further reduce mortality risk may have been a step too far.

The true CV benefit with SGLT2-inhibition could be benchmarked against the only other novel therapy shown to reduce mortality in a well-treated HF population—the angiotensin receptor-neprilysin inhibitor sacubitril-valsartan, which works by augmenting the natriuretic peptide system and simultaneously inhibiting the renin–angiotensin–aldosterone system [33]. Dapagliflozin and empagliflozin both showed greater relative and absolute risk reduction in primary composite outcome of CV death or HF hospitalisation compared to sacubitril-valsartan (PARADIGM-HF) in comparable cohorts of HF patients [34]. The benefit seen with SGLT2-inhibitors was primarily driven by superior reductions in HF hospitalisation. In fact, the effect size seen in the SGLT2-inhibitor trials may have been undermined by the relatively older and sicker (larger proportion of NYHA III/IV) cohort and a significantly shorter median follow-up time.

### HF with preserved ejection fraction – are SGLT2-inhibitors the holy grail?

HF with preserved ejection fraction (HFpEF) makes up approximately half of the HF population. Both HFrEF and HFpEF have similar prognoses but very different pathophysiology [35]. HFrEF is due to impaired emptying of the left ventricle (LV) from systolic dysfunction, perpetuated by systemic neurohormonal activation. HFpEF on the other hand is largely due to LV diastolic dysfunction, either passively from increased myocardial stiffness or actively from impaired relaxation [36]. The lack of efficacy seen with ACE-inhibitors/ARBs [37–39], MRAs [40] and sacubitril / valsartan [41] in HFpEF suggest that excessive loading is not a key pathogenic mechanism or adverse adaptation in this HF phenotype. Increasing evidence points to abnormalities at the cellular level (inflammation, myocardial energetics, calcium handling and extracellular matrix composition) as potential targets for HFpEF management [42]. As we will discuss in the subsequent sections, SGLT2-inhibitors have unique properties that could specifically address these molecular changes particularly around calcium handling and myocardial energetics.

Several large dedicated HFpEF studies; Empagliflozin Outcome Trial in Patients with Chronic Heart Failure with Preserved Ejection Fraction (EMPEROR-Preserved) (NCT03057951) and Dapagliflozin Evaluation to Improve the Lives of Patients with Preserved Ejection Fraction Heart Failure (DELIVER) (NCT03619213), are nearing completion and will characterize SGLT2-inhibitor benefit in this cohort.

### Cardiorenal syndrome – an unmet need in HF

The heart and kidney are inextricably linked, where direct or indirect effects of one dysfunctional organ can initiate and perpetuate the combined disorder of both organs, and is often referred to as the cardiorenal syndrome [43]. Up to 60% of patients with HF have co-morbid chronic kidney disease (CKD), thus increasing their risk of mortality compared with patients with just HF alone [44, 45]. Besides cardioprotection, the renal protective effects of SGLT2-inhibitors are derived from multiple converging pathways related to its primary mechanism of action; natriuresis and glucosuria.

In patients with T2DM, the increased filtered glucose elicits upregulation of SGLT1 and SGLT2 expression in the proximal convoluted tubule of the nephron. As a consequence of the increased glucose (and sodium) absorption by these transporters in the proximal part of the nephron, there is reduced sodium delivery distally to the macula densa causing afferent renal arteriolar dilatation and hyperfiltration. Excessive hydrostatic damage to the glomerulus from hyperfiltration is thought to be the main driver for diabetic nephropathy [46]. By inhibiting sodium resorption in the proximal nephron thereby increasing distal delivery, SGLT2-inhibitors can restore tubuloglomerular feedback and normalise renal blood flow. The preferential effect of SGLT2-inhibition on the afferent, rather than efferent arteriole, may explain why renal function returns to baseline after an initial dip in estimated glomerular filtration rate (eGFR), with stability over time (positive eGFR slope) [47, 48]. In comparison, RAAS-inhibitors slow renal disease progression but eGFR either stabilises after the initial dip or slowly declines over a similar period (negative eGFR slope) [49, 50]. In addition, cardiorenal syndrome severely limits the available options of HF medications (ACE-inhibitors/ARBs, MRAs and ARNIs), all of which cumulatively increase risk of acute kidney injury (AKI) and hyperkalaemia in a dose-dependent manner. As Lam et al. point out, cardiorenal syndrome remains one of the key unmet needs in the management of HF, which could be directly addressed by SGLT2-inhibitors [51].

Following consistently positive effects on renal endpoints in early trials, the Evaluation of the Effects of Canagliflozin on Renal and Cardiovascular Outcomes in Participants with Diabetic Nephropathy (CREDENCE) trial was the first dedicated renal endpoint study in patients with diabetic kidney disease, using canagliflozin. Of the 4401 patients, all had a diagnosis of chronic kidney disease, 50.4% also had established CV disease and 16% had baseline HF [52]. Canagliflozin delayed renal disease progression (HR 0.60; 95% CI 0.48–0.76) and reduced risk of end-stage kidney disease (HR 0.68; 95% CI 0.54–0.86) [52]. Risk of hospitalisation from HF was also significantly reduced by 39%, consistent with results from previous CVOTs. There was no increase in incidence of AKI or hyperkalaemia with SGLT2 inhibition.

A Study to Evaluate the Effect of Dapagliflozin on Renal Outcomes and Cardiovascular Mortality in Patients With Chronic Kidney Disease (DAPA-CKD) provides further affirmation for SGLT2-inhibitor use in both diabetic and non-diabetic CKD aetiologies. Dapagliflozin reduced the risk of worsening renal function or death from kidney failure by 44%, hospitalisation for HF or CV death by 29% and all-cause mortality by 31% (all p < 0.05) – regardless of T2DM status [53]. Results from EMPA-KIDNEY are keenly awaited.

### Are SGLT2-inhibitors a diabetes drug or a drug for HF and CKD?

Despite multiple guideline recommended disease-modifying therapies, patients with HF continue to have a poor prognosis [54]. Results from recent CV and renal outcome trials are very promising highlighting SGLT2-inhibitors' potential to treat T2DM, CV disease, HF and CKD—conditions that are invariably linked by the common CV risk factor profile [18, 22, 23, 52]. It has also become evidently clear that the cardiorenal benefits of SGLT2-inhibitors are independent of its modest reductions in conventional risk factors (HbA1c, blood pressure, cholesterol), T2DM status and renal function.

The question  remains: Should cardiologists and nephrologists be proactively prescribing SGLT2-inhibitors to all patients with CV and renal disease?

From a CV perspective, there have now been analyses of three patient groups; 1) primary prevention cohort (part of CANVAS and DECLARE TIMI-58), 2) secondary prevention cohort (EMPA-REG OUTCOMES, VERTIS CV, part of CANVAS and DECLARE TIMI-58) and 3) HF cohort (DAPA-HF and EMPEROR-Reduced). As the CV risk profile increases, so does the apparent ‘efficacy’ of SGLT2-inhibition on CV mortality and HF hospitalisation. However, as seen in EMPEROR-Reduced, there is a signal that there may be an upper limit of HF severity beyond which the mortality benefits of SGLT2-inhibition plateaus, whilst morbidity benefits (reduced hospitalisations) remain robust throughout the entire spectrum of disease risk and severity.

So far only canagliflozin has received regulatory approval for use in patients with T2DM and CKD following data from the CREDENCE trial [55]. In patients with non-diabetic nephropathy, the answer is slowly emerging, especially following very encouraging results from DAPA-CKD, resulting in it being granted fast-track designation by the FDA. [56]. This highlights the urgent clinical need to slow down CKD progression and improve quality of life as well as life expectancy in this expanding cohort. Current evidence indicate that the renoprotective benefits conferred may just be the most striking effect of this drug class thus far—perhaps as a consequence of the very limited treatment options available for CKD, unlike in HF.

### Mechanistic trials in SGLT2-inhibitors—more questions than answers

A characteristic feature of the pathophysiology of HF is the activation of compensatory pathways; however, sustained activation of the neurohormonal system (sympathetic nervous system and renin–angiotensin–aldosterone system) results in maladaptive remodelling of the ventricles and myocardial injury, which perpetuate the disease state. Being able to reverse this remodelling is an important determinant of long-term severity of HF and mortality [57]. Previous HF therapies have all unequivocally exhibited reverse remodelling effects on the failing heart [58–60]. It was therefore sensible that the main premise behind the first batch of mechanistic studies of SGLT2-inhibitors were based on the hypothesis that they too could induce reverse remodelling owing to the diuretic and BP lowering properties, which should improve ventricular loading in the dysfunctional ventricle.

The Research Into the Effect of SGLT2 Inhibition on Left Ventricular Remodeling in Patients With Heart Failure and Diabetes Mellitus (REFORM) trial was the first to try to determine the mechanistic effect of SGLT2-inhibitors specifically in the HF population. At 1 year, no significant difference in LV remodelling (LV end systolic volume, end diastolic volume, LV mass index or EF), as assessed by cardiac magnetic resonance imaging was seen with dapagliflozin therapy [61]. The absence of effect on remodelling was unexpected given the striking improvements in HF outcomes. There are two possible reasons why dapagliflozin therapy was neutral on LV remodelling. First, the majority of patients in the REFORM trial were on ACEi/ARBs (89%), beta-blockers (82%) and MRA (41%), which have potent effects on ventricular loading. The addition of an SGLT2-inhibitor, with only modest diuretic and BP effects, would thus confer little incremental benefit on haemodynamic load and parameters of LV remodelling. Similar conclusions can also be drawn from the DEFINE-HF Trial, where dapagliflozin did not significantly reduce NT-proBNP levels, a biomarker of ventricular end diastolic pressure, over 12 weeks, as compared to placebo [62]. Second, improvement in LV remodelling seen with SGLT2-inhibitors appear to usually occur in the early stages of the disease spectrum. Post-hoc exploratory analyses from the REFORM trial suggest dapagliflozin reduced LV volumes and indexed LV mass in patients with LVEF ≥ 45%, with no interaction seen in other key secondary outcomes [63]. These findings are consistent with improvements in HF outcomes in the CVOTs, in which a large proportion of patients probably had early, occult HF. Other mechanistic trials such as the EMPA-HEART and DAPA-LVH trials support this hypothesis, demonstrating reductions in LV mass in patients with T2DM and LV hypertrophy without LV dysfunction or symptoms of HF [64].

Putting everything together, there is a definite clinical benefit from SGLT2-inhibitor therapy particularly in the context of hard HF outcomes, however the mechanism of that effect remains unclear (Table 1). The glucose-lowering and diuretic effects from its primary mode of action on the nephron, although important, simply cannot explain the speed and magnitude of benefit seen in the large outcome trials. Early mechanistic trials on conventional markers of LV remodelling have been inconclusive, raising the possibility that SGLT2-inhibitors act in a novel way that is not reflected by changes in LV remodelling as we currently understand them. In the following sections we explore new and emerging hypotheses into the molecular changes brought about by SGLT2-inhibition and their potential role in treating HF.

**Table 1:** Overview of recent SGLT2-inhibitor clinical trials design and results

Trial Name	Drug	Duration (median)	Cohort	Primary outcome	Key secondary outcomes
** Cardiovascular Outcome Trials**					
EMPA-REG Outcome [18]	Empagliflozin vs. placebo	3.1 years	n = 7020; T2DM with established CVD	3P-MACE (HR 0.86; 95% CI 0.74–0.99)	- 3P-MACE + hospitalisation for UA (HR 0.89; 95% CI 0.78 to 1.01) - CV death (HR 0.62; 95% CI 0.49 to 0.77) - HHF (HR 0.65; 95% CI 0.50 to 0.85) - CV death/HHF (HR 0.66; 95% CI 0.55 to 0.79) - Death from any cause (HR 0.68; 95% CI 0.57 to 0.82)
CANVAS [22]	Canagliflozin vs. placebo	2.4 years	n = 9734; Poorly controlled T2DM plus i) age 30 + and history of symptomatic atherosclerotic CVD or ii) age 50 + and high risk of CVD	3P-MACE (HR 0.86; 95% CI 0.75–0.97)	- CV death (hazard ratio, 0.87; 95% CI 0.72 to 1.06) - Progression of albuminuria (30% increase) (HR 0.73; 95% CI 0.67 to 0.79) - CV death/HHF (HR 0.78; 95% CI 0.67 to 0.91)
DECLARE-TIMI 58 [23]	Dapagliflozin vs. placebo	4.2 years	n = 17,160; age 40 + with T2DM and either history or high risk of atherosclerotic CV events	3P-MACE (HR 0.93; 95% CI 0.84–1.03) CV death/HHF (HR 0.83; 95% CI 0.73–0.95)	- > 40% reduction in eGFR/new ESRD/renal death/CV death (HR 0.76; 95% CI 0.67–0.87) - Death from any cause (HR 0.93; 95% CI 0.82–1.04)
VERTIS CV [28]	Ertugliflozin vs. placebo	6.1 years	n = 8246; T2DM and established ASCVD	3P-MACE (HR 0.97; 95% CI 0.85–1.11)	- CV death/HHF (HR 0.88; 95% CI 0.75–1.03) - HHF (HR 0.70; 95% CI 0.54–0.90) - Progression of renal disease (HR 0.81; 95% CI 0.63–1.04)
**Heart Failure Outcome Trials**					
DAPA-HF [31]	Dapagliflozin vs. placebo	1.5 years	n = 4744; HFrEF (LVEF < 40%); NTproBNP > 400–600 (depending on criteria); with or without T2DM	Time to first occurrence of CV death/ HHF/ urgent HF visit (HR 0.74; 95% CI 0.65–0.85)	- CV Death/HHF (HR 0.75; 95% CI 0.65–0.85) - ≥ 50% sustained reduction in eGFR/ reaching ESRD/renal death (HR 0.71; 95% CI 0.44–1.16) - KCCQ (HR 1.18; 95% CI 1.11–1.26) - Death from any cause (HR 0.83; 95% CI 0.71–0.97)
EMPEROR-Reduced [32]	Empagliflozin vs. placebo	16 months	n = 3730; HFrEF (LVEF ≤ 40%; NYHA II-IV); NTproBNP > 600–5000 (specific criteria based on diagnosis of AF and EF); with or without diabetes	Time to first occurrence of CV death/ HHF (HR 0.75; 95% CI 0.65–0.86)	- CV death (HR 0.92; 95% CI 0.75–1.12) - First HHF (HR 0.69; 95% CI 0.59–0.81) - HHF (HR 0.70; 95% CI 0.58–0.85) - Decline in eGFR (1.73 ml/min/1.73m^2^/year slower decline in treatment arm; 95% CI 1.10–2.37 - Death from any cause (HR 0.92; 95% CI 0.77 to 1.10)
DELIVER [65] (Currently recruiting – est completion June 2021)	Dapagliflozin vs. placebo	2.75 years	n = 6100; HFpEF (LVEF > 40%); Elevated NT-proBNP; Ambulatory and hospitalised patients	Time to first occurrence of CV death/ HHF/ urgent HF visit	- KCCQ - Worsening NYHA class - Total number of CV death or HHF - Time to death from any cause
EMPEROR-Preserved [66] (est completion Nov 2020)	Empagliflozin vs. placebo	3.2 years	n≈5988; HFpEF (LVEF > 40%) + structural heart disease; NTproBNP > 300; with or without diabetes	Time to first occurrence of CV death/ HHF	- CV death - HHF - All-cause hospitalisation - Change in KCCQ - RRT or sustained reduction of ≥ 40% eGFR - All-cause mortality - Onset of DM
**Mechanistic / Biomarker Trials**					
REFORM [61]	Dapagliflozin vs. placebo	12 months	n = 58; T2DM; stable HFrEF (LVEF ≤ 45%) NYHA class I-III; on furosemide ≤ 80 mg or other loop diuretics; eGFR ≥ 45	Change in indexed LVESV (2.49 mL/m2; 95% CI -6.30 to 11.28)	- LVMi (2.5 g/m2; 95% CI -3.95 to 8.95) - LVEF (0.69%; 95% CI -3.32 to 4.69) -LVEDV (indexed; 3.9 mL/m2; 95% CI -7.05 to 14.85) - Weight (-2.26 kg; 95% CI -4.83 to 0.31) - SBP (-4.7 mmHg; 95% CI -14.51 to 5.11) - Haematocrit (2.89%; 95% Cl 1.14 to 4.64) - Loop diuretic dose (-29.06 mg; 95% CI -42.17 to -15.95)
RECEDE-CHF [17]	Empagliflozin vs. placebo	6 weeks	n = 23; T2DM and HF (NYHA II-III; LVEF < 50%); eGFR ≥ 45 ml/min/1.73m^2^; median NT-proBNP 2381; on furosemide or equivalent loop diuretic therapy	Mean change in 24-h urinary volume at 6 weeks (545 ml, 95% CI 136–954)	- 24-h urinary sodium excretion (7.85 mmol/L; 95% CI -2.43 to 6.73) - Electrolyte-free water clearance (312 ml; 95% CI 26–598) - Fractional clearance of sodium (0.11%; 95% CI -0.22 to 0.44) - Change in urine creatinine (-1.66 mmol/L; 95% CI -3.07 to -0.25) - Change in NTproBNP (283.4 pg/ml; 95% CI -835.8 to 1402.3)
DAPA-LVH [67]	Dapagliflozin vs. placebo	12 months	n = 66; T2DM; BMI ≥ 23; BP < 145/90 mmHg; LV hypertrophy	Change in indexed LVM (-2.82 g; 95% CI -5.13 to -0.51)	- LVMi (-0.20 g/m2; 95% CI -1.21 to 0.80) - LVEF (0.79%; 95% CI − 1.14 to 2.72) - EDV (-1.59mLS; 95% CI -7.06 to 3.87) - ESV (-1.12mLs; 95% CI -3.50 to 1.25) - Ambulatory SBP (-3.63 mmHg; 95% CI -6.44 to -0.82) - Abdominal obesity (-609.76cm^3^; 95% CI -948.13 to -271.28) - Weight (-3.77 kg; 95% CI -4.92 to -2.61) - Haematocrit (2.90%; 95% CI 1.84 to 3.96) - NT-proBNP (-103.68 pg/mL; 95% CI -326.90 to 119.54)
EMPA HEART Cardio-Link 6 [64]	Empagliflozin vs. placebo	6 months	n = 97; T2DM; established CVD (MI ≥ 6 months, or coronary revascularization ≥ 2 months)	Change in indexed LVM (-3.35 g/m2; 95% CI -5.9 to -0.81)	- LVEDV (− 0.9 mL/m2; 95% CI -8.5 to − 6.7) - ESV (− 2.2 mL/m2; 95% CI -7.3 to 2.8) - LVEF (2.2%; 95% CI -0.2 to 4.7) - NT-proBNP (7.4 pg/mL; 95% CI − 10.3, 25.1)
DEFINE-HF [62]	Dapagliflozin vs. placebo	12 weeks	n = 263; HFrEF (LVEF ≤ 40%, and NYHA class II-III) with or without T2DM	i) average of 6- and 12-week mean NT-proBNP (HR 0.95; 95% CI 0.84 to 1.08) ii) ≥ 5-point increase in average of 6- and 12-week KCCQ-OS or ≥ 20% reduction in average of 6- and 12-week NT-proBNP (OR 1.8, 95% CI 1.03 to 3.06)	- KCCQ 6 weeks (OR 1.8; 95% CI 1.04 to 3.12) and 12 weeks (OR 1.7; 95% CI 0.98 to 3.1) - ≥ 20% reduction NT-proBNP at 6 weeks (OR 1.1; 95% CI 0.6 to 1.9) and 12 weeks (OR 1.9; 95% CI 1.09 to 3.31) - ≥ 20% reduction in BNP at 6 weeks (OR 1.5; 95% CI 0.9 to 2.7) and 12 weeks (OR 2.0; 95% CI 1.1 to 3.4) - 6MWT—adjusted distance at week 12 (304 m vs 301 m) - Mean weight reduction of -1.33 kg without T2DM Dapa vs. placebo (95% CI -2.41 to -0.23 kg) - HHF or urgent HF visits (HR 0.84; 95% CI 0.35 to 1.97)
EMPA-TROPISM [68] (est completion Dec 2020)	Empagliflozin vs. placebo	6 months	n = 84; stable HFrEF (LVEF < 50%) NYHA II-III); with or without T2DM	Changes in LVESV/LVEDV	- LVEF index - VO_2_ Consumption - 6MWT - KCCQ-12
**Renal Outcome Trials**					
CREDENCE [52]	Canagliflozin vs. placebo	2.6 years	n = 4401; T2DM; CKD (eGFR 30 to < 90 ml/minute/1.73 m^2^); albuminuria (UACR > 300 to 5000); on ACEi/ARB therapy	ESRD/ serum creatinine × 2 baseline (30 + days)/ renal or CV death (HR 0.70; 95% CI 0.59 to 0.82)	- CV death/HHF (HR 0.69; 95% CI 0.57 to 0.83) - CV death/ MI/ stroke (HR 0.80; 95% CI 0.67 to 0.95) - HHF (HR 0.61; 95% CI 0.47 to 0.80) - CV death (HR 0.78; 95% CI 0.61 to 1.00) - Death from any cause (HR 0.83; 95% CI 0.68 to 1.02) - CV death/ MI/ stroke/ hospitalization for HF or UA (HR 0.74; 95% CI 0.63 to 0.86)
DAPA-CKD [69]	Dapagliflozin vs. placebo	2.4 years	n = 4304; with or without diabetes; eGFR ≥ 25 and ≤ 75 ml/min/1.73m^2^; UACR ≥ 200 or ≤ 5000 mg/g; maximum tolerated daily dose of ACEi or ARB	≥ 50% decline in eGFR/reaching ESRD/CV death/renal death (HR 0.61; 95% CI 0.51–0.72)	- HHF/ CV death (HR 0.71; 95% CI 0.55–0.92) - Death from any cause (HR 0.69; 95% CI 0.53–0.88) - ≥ 50% decline in eGFR/reaching ESRD/renal death (HR 0.56; 95% CI 0.45–0.68)
EMPA-Kidney [70] (est completion June 2022)	Empagliflozin vs. placebo	3.1 years	n≈6000; CKD + risk of kidney disease progression (depending on criteria); g on ACEi or ARB therapy	Time to first occurrence of: (i) Kidney disease progression (ESRD, sustained decline in eGFR to < 10 mL/min/1.73m^2^, renal death, decline of ≥ 40% in eGFR) or (ii) Cardiovascular death	Time to: - HHF or CV death - All-cause hospitalisations - Death from any cause - First occurrence of kidney disease progression - CV death - CV death or ESRD

3P-MACE, Composite of: CV Death; non-fatal MI; non-fatal Stroke); ASCVD, atherosclerotic cardiovascular disease; UA, unstable angina; LVEF, left ventricle ejection fraction; ESRD, end Stage Renal Disease; KCCQ, Kansas City Cardiomyopathy Questionnaire; 6MWT, 6-min walk test; ITT, intention to treat analysis; LVSD, left ventricular systolic dysfunction; LV, left ventricle; CVD, cardiovascular disease; LVEDV, LV end-diastolic volume; ESV, End-systolic volume; EDV, end diastolic volume; BIA, Bioelectrical impedence Analysis; CPET, Cardio-pulmonary Exercise Testing; LVM, LV mass; CMRI, cardiac magnetic resonance imaging; LVMi, BSA indexed LVM; CKD, chronic kidney disease; ACEi, angiotensin-converting–enzyme inhibitor; ARB, angiotensin-receptor blocker; UACR, urine albumin-to-creatinine ratio; RRT, renal replacement therapy

### Calcium and the cardiomyocyte

The ionic balance within the cardiomyocyte is finely modulated by a variety of ion pumps on the cell surface membrane which interact via multiple overlapping pathways. Calcium (Ca^2+^), is a key ion involved in excitation–contraction coupling, cardiac rhythmicity, also acting as a second messenger in regulating gene transcription for myocyte hypertrophy and other pathological remodelling pathways [71].

**Fig. 3:**
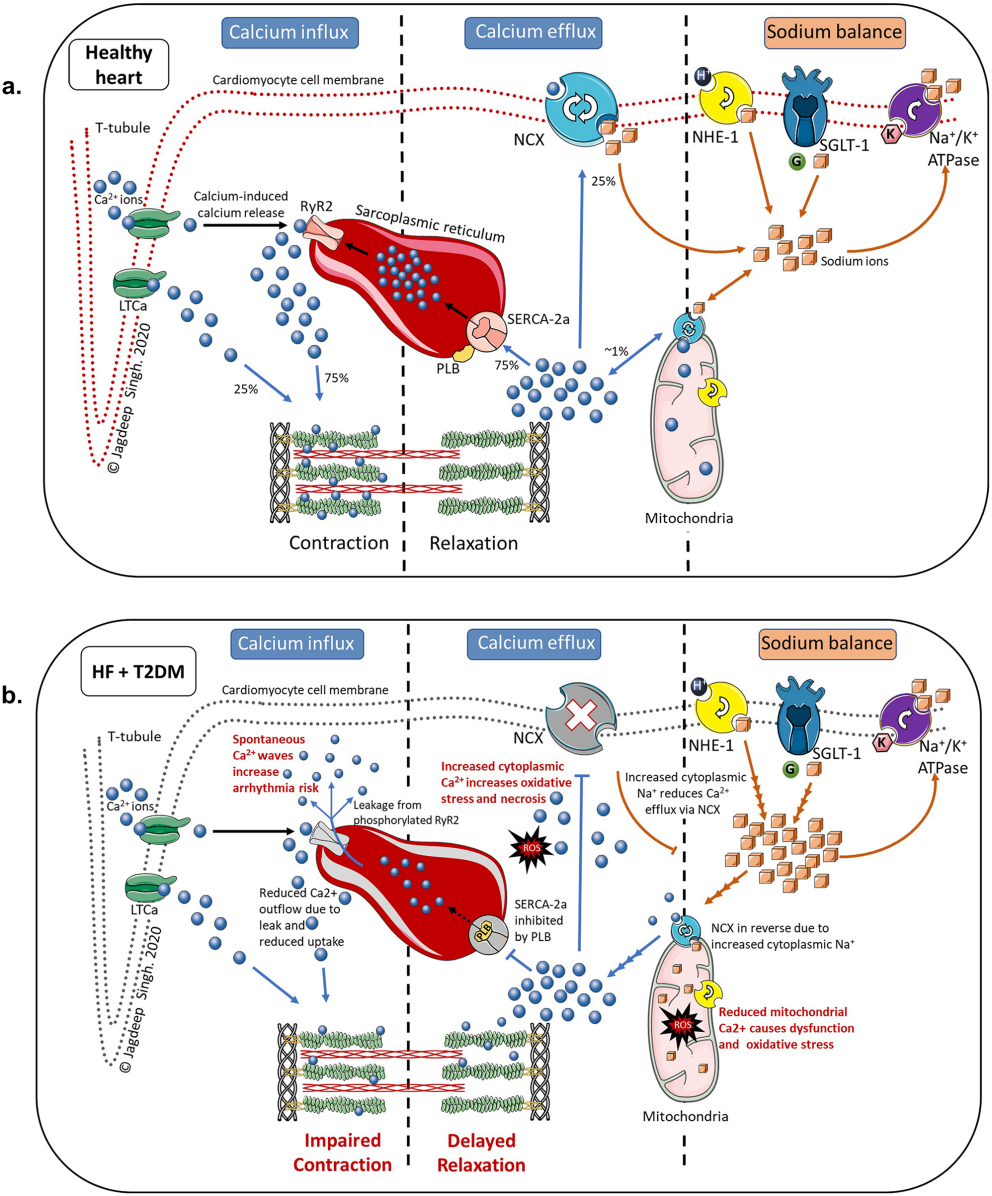
Schematic representation of sodium and calcium ion balance in the cardiomyocyte. Figure 2a: Ion balance in a healthy heart. Figure 2b: Abnormal calcium and sodium balance as a consequence of heart failure and type 2 diabetes Abbreviations: Ca^2+^-calcium; K^+^-potassium; LTCa-L-type calcium channel; Na^+^ -sodium; Na^+^/K^+^ ATPase-sodium–potassium adenosine triphosphatase pump; NCX-sodium-calcium exchanger; NHE-1-sodium-hydrogen exchanger subtype-1; PLB-phospholamban; ROS-reactive oxygen species; RyR2-ryanodine receptor 2; SERCA2a- sarcoplasmic reticulum calcium adenosine triphosphatase subtype 2a; SGLT-1-sodium-glucose cotransporter subtype 1; Blue spheres: calcium ions; Orange cubes: sodium ions; Green sphere: glucose molecule: Pink hexagon: potassium ion

Action potentials generated at the cell surface trigger the opening of voltage-dependent L-type Ca^2+^ channels facilitating Ca^2+^ influx into the cardiomyocyte where it then binds to ryanodine R2 receptors (RyR2) on the sarcoplasmic reticulm (SR) to release intrasarcoplasmic Ca^2+^. This process (called calcium-induced calcium release) amplifies the cytoplasmic Ca^2+^ content, which then bind to cardiac troponin, triggering muscle contraction. At the end of systole, the majority of Ca^2+^ ions are recycled back to the SR for storage via the SR Ca^2+^-ATPase (SERCA2a), while the rest are extruded extracellularly via the sodium-calcium exchanger (NCX) [72] (Fig. 3).

HF and T2DM cause structural and functional changes to these pumps, and in doing so, disrupt the finely-balanced calcium homeostasis. There is increased activity of phospholamban which inhibits SERCA2a resulting in reduced Ca^2+^ reuptake into SR. This prolongs the relaxation phase (causing diastolic dysfunction) and reduces SR Ca^2+^ content available for use in the next contraction cycle (cauing systolic dysfunction). Likewise, phosphorylation of RyR2 pumps cause diastolic leakage of Ca^2+^ from the SR further reducing its storage capacity and increasing arrhythmogenicity due to increased cytosolic Ca^2+^ during diastole [73]. Additionally, there is also increased expression of SGLT1 and the sodium-hydrogen exchanger 1 (NHE1) on the cardiomyocyte cell membrane which increases intracytoplasmic sodium (Na^+^) concentrations [74, 75]. This reduces the efficiency of NCX pumps on the cell surface as well as those on mitochondria (which require low intracytoplasmic Na^+^ concentrations), resulting in increased cytoplasmic Ca^2+^ and reduced mitochondrial Ca^2+^ leading to myocyte hypertrophy, increased oxidative stress and accelerated cell death [76, 77] (Fig. 3).

SGLT2 is not expressed on the heart [78], yet there is strong pre-clinical evidence that SGLT2-inhibitors influence Ca^2+^ handling by modulating intracytoplasmic Na^+^ in the cardiomyocyte. This is supported by evidence that empagliflozin improves SERCA2a efficiency [79] and reduces RyR2-dependent Ca^2+^ leak [80, 81]. Additionally, empagliflozin may also preserve mitochondrial function and reduce oxidative damage [82]. SGLT2-inhibitors achieve this by inhibiting NHE1 (SGLT2-inhibitors inactivate NHE1 by binding to its Na^+^ binding site) and possibly SGLT1 as well (all SGLT2-inhibitors have intrinsic SGLT1 blocking ability – albeit to different degrees) [77, 83, 84].

Interestingly, T2DM is not the only trigger for SGLT1 upregulation in the heart; there is a 31% increase in SGLT1 expression seen in obese HF patients *without* T2DM [75]. Furthermore, preclinical data on empagliflozin shows its effects on myocyte Ca^2+^ and Na^+^ is independent of extracellular glucose levels and SGLT2 activity [85]. These findings could possibly explain the equal benefit of dapagliflozin in patients with or without T2DM in the DAPA-HF and EMPEROR-Reduced trials. Nevertheless, the only way to truly determine the effect of SGLT2-inhibiton on myocardial ionic homeostasis is by performing a dedicated clinical trial.

## Powering the heart

The healthy heart utilises a variety of fuels to power its function. Under normal conditions, mitochondrial oxidative phosphorylation accounts for 95% of myocardial energy demands, whilst only 5% is met by glycolysis and TCA cycle. Between 70%—90% of the ATP generated by the heart is derived from free fatty acids (FFA) and the remainder from glucose and other substrates such as ketones, branch chain amino acids and lactate [86]. The heart can rapidly alter its fuel mix between these substrates depending upon the workload, perfusion and substrate bioavailability [87]. There are, however, important differences between these fuels; FFA requires more oxygen per ATP molecule produced compared to the more oxygen efficient glucose. However, each molecule of the energy dense FFA produces more ATP than that of glucose. Interestingly, ketones are more efficient than both, producing more energy with less oxygen compared to glucose and FFA respectively – leading to it being dubbed as a ‘super fuel’ [88].

FFAs are the primary energy source for the heart  under most conditions, although its proportion of the overall fuel mix changes situationally. For example in the post prandial state, with higher circulating glucose and insulin, there is increased glucose uptake through myocardial GLUT proteins [86]. Following exercise when levels are raised, more lactate is used for energy production. Similarly, when ketone levels are raised it becomes the preferred fuel; and the heart has been shown to be able to oxidise more ketones per unit of mass than any other organ [89]. In contrast, during high intensity exercise (physiological hypoxemia) or ischaemia (pathological) there is a shift toward the more oxygen-efficient glucose and glycolytic ATP production [88].

**Fig. 4:**
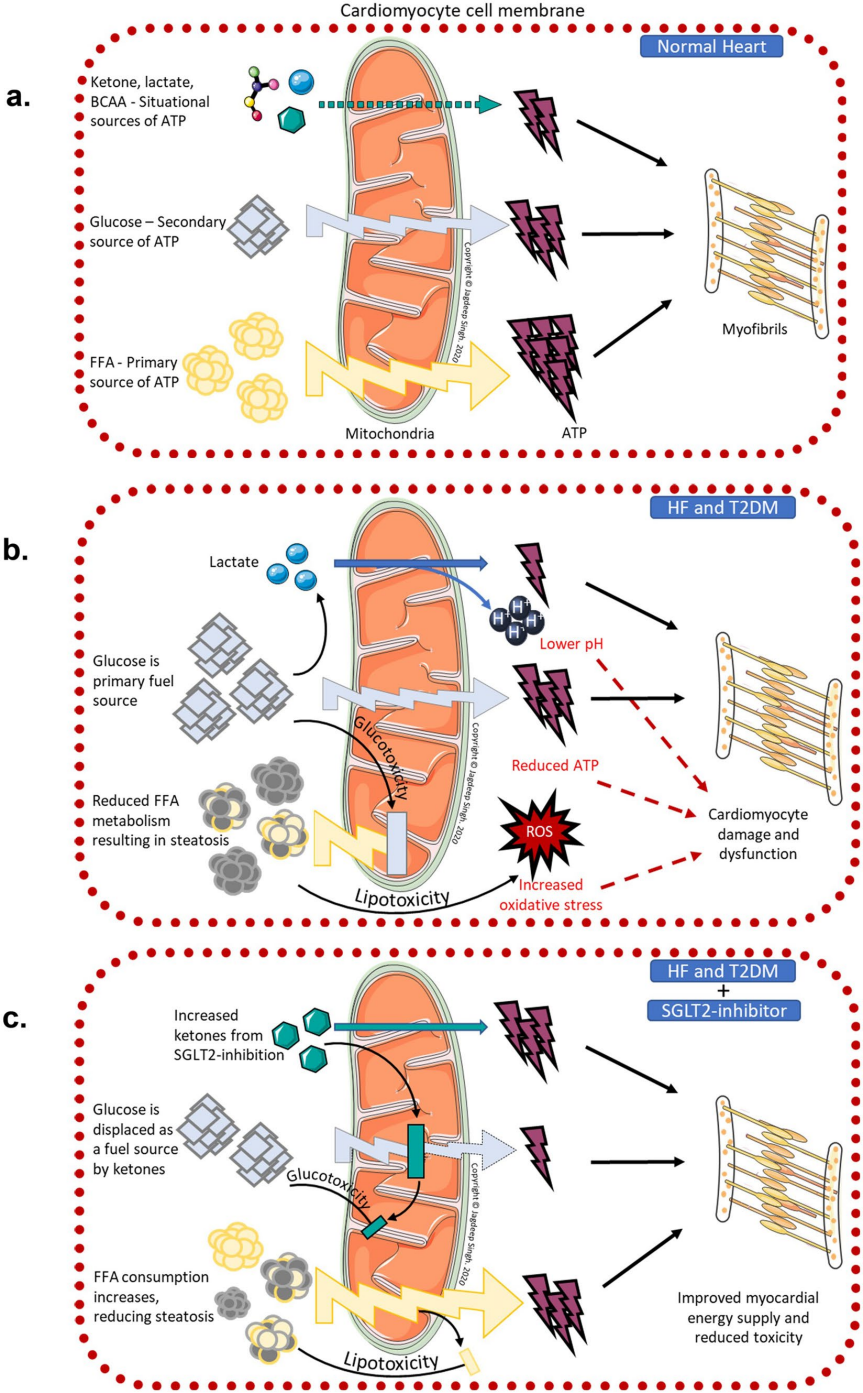
Schematic representation of myocardial energy consumption. Figure 3a: Myocardial energetics in healthy heart under resting conditions. Figure 3b: Myocardial energetics as a consequence of heart failure and type 2 diabetes. Figure 3c: Changes in myocardial energetics with the use of SGLT2-inhibitor therapy in patients with heart failure and type 2 diabetes Abbreviations: ATP-adenosine triphosphate; BCAA-branch chain amino acid; FFA-free fatty acid; HF-heart failure; ROS-reactive oxygen species; SGLT2- sodium-glucose cotransporter subtype 2; T2DM- type 2 diabetes mellitus

This shift in metabolism to favour glucose over FFA is a rapid response mechanism to acute changes, however, just like neurohormonal activation in the context of ventricular loading, if left unchecked it can result in ‘metabolic remodelling’ leading to cardiomyocyte dysfunction. Indeed, it is the most consistent metabolic change seen in animal and clinical studies of HF. A chronic overreliance on glucose results in suppression of genes related to FFA beta-oxidation pathways including their upstream regulators such as PPAR-alpha [90]. Downregulation of FFA oxidation capacity causes intracytoplasmic FFA accumulation, resulting in cardiac steatosis and lipotoxicity from reactive oxygen species [91]. Additionally, there will also be a large energy deficit due to the inability of glucose to produce nearly as much ATP as FFA oxidation. Together, this metabolic remodelling results in increased cellular toxicity and an energy deficit, culminating in cardiomyocyte dysfunction [90] (Fig. 4).

On the other hand, insulin resistance and increased proinflammatory cytokinesis (TNF and IL-6) which are hallmarks of T2DM, also induce lipolysis thereby increasing FFA delivery to the cardiomyocyte and worsening lipotoxicity [92]. As a further complication, insulin resistance reduces glucose delivery into the cardiomyocyte from insulin-sensitive GLUT4 (predominant route of glucose entry into cardiomyocyte) and GLUT8, forcing the heart to rely on the lower capacity (and insulin-independent) GLUT1 and SGLT1 routes. This exacerbates the energy deficit already present from lipotoxicity, thereby accelerating cardiomyocyte dysfunction [90, 93].

Given the effects of the various fuel substrates on cardiac efficiency and potentially even cellular dysfunction, optimizing myocardial energetics could be a key therapeutic target for patients with HF and T2DM. SGLT2-inhibitors are uniquely poised to fill this area of need as they have consistently shown to increase plasma ketone levels by increased hepatic synthesis from lipolysis and reduced renal loss [94]. (Fig. 2) Indeed, a recent porcine model of post infarct HF found metabolic remodelling with reduced FFA metabolism and anaerobic glycolysis resulted in a significant energy deficit. However, treatment with empagliflozin was able to switch myocardial metabolism away from anaerobic glycolysis toward utilisation of ketones, FFA and branch chain amino acids. The improved energetics resulted in higher myocardial ATP content and reduced adverse LV remodelling, enhanced LV function and reduced neurohormonal activation at 2 months post infarct [89]. Clinically, Nielsen and colleagues showed that an infusion of ketones bodies in patients with HF resulted in increased cardiac output and improved EF with an associated increase in heart rate and myocardial oxygen consumption. This was a small, proof of concept trial in HF patients without T2DM where measurements were taken acutely following ketone infusion, administered in low dose insulinemic euglycaemic clamp conditions, acheiving fairly high levels of ketonemia [95]. It is yet unclear what the effects of a more modest, chronic ketonemia, as seen in SGLT2-inhibition, will be. There are ongoing efforts to determine the clinical effect of SGLT2-inhibition on myocardial energetics in the context of HF [96]. These studies will help determine if the future of managing HF will include metabolic modulation to improve myocardial energetics just as neurohormonal modulation to improve ventricular loading forms the backbone of HF therapeutics today.

### SGLT2-inhibitor induced erythropoiesis

SGLT2-inhibitors increase haemoglobin and haematocrit levels. Initially these observations were attributed solely to haemoconcentration from its diuretic effect [97]. We now know that although there is an initial rise in urinary volume, this plateaus after a few weeks but haematocrit continues to rise well beyond that, suggesting a different mechanism driving this effect [98]. There is strong evidence demonstrating SGLT2-inhibition inducing erythropoiesis; a 12-week study reported increased haematocrit, haemoglobin, reticulocyte count and erythropoietin (EPO) with dapagliflozin [99], with similar results in a trial using empagliflozin in patients with T2DM and ishcaemic heart disease [100].

The mechanism behind these observations have yet to be elucidated, however there are a few hypotheses. EPO is synthesized in the renal cortex by EPO-producing fibroblasts. Patients with T2DM have increased filtration of glucose resulting in upregulation of SGLT1 and SGLT2 in the PCT to increase glucose resorption capacity, however this is a net energy-consuming process. The resulting relative cortical hypoxia and increased oxidative stress from higher energy demands of these transporters in the renal cortex causes the cortical fibroblasts to undergo transformation into myofibroblasts which no longer produce EPO. SGLT2-inhibitors block these transporters, thereby reducing energy demands—the cortical injury is reduced, the transformation reverses and EPO production capacity is restored. Additionally, SGLT2-inhibition increases sodium delivery to the distal portions of the nephron which causes upregulation of medullary sodium transporters in the loop of henle and terminal nephron resulting in relative medullary hypoxia, which stimulates erythropoiesis. It has also been suggested that increased plasma ketone levels, which are associated with SGLT2-inhibitor use, directly contribute to EPO synthesis [100, 101].

There is evidence that erythropoiesis-stimulating agents (in the non-HF population) have beneficial cardiac effects including increased survival following ischaemia–reperfusion injury, reduced apoptosis, increased angiogenesis and improved myocardial contractility [102–104]. However, previous trials using various erythropoiesis-stimulating agents in patients with HF have been underwhelming and in the case of darbepoetin alfa, there was no effect on HF outcomes, but an increased risk of thromboembolic events [105]. Indeed, this was also an initial concern with SGLT2-inhibitor therapy [106] but that has not been borne out with more recent trials and real-world data [18, 22, 23, 107]. In fact, a mediation analysis of the EMPA-REG OUTCOME showed changes in haemoglobin and haematocrit were responsible for approximately 50% of the risk reduction in CV death in that trial [108]. Perhaps SGLT2-inhibitors induce a more physiological effect, by restoring favourable renal physiology, which is salutary to the heart compared to exogenous stimulation of erythropoiesis by other agents.

### Modulation of the sympathetic nervous system

The sympathetic nervous system is closely linked to the pathophysiology of HF and T2DM. Hypoperfusion from HF results in sympathetic activation, however persistently elevated sympathetic activity results in ventricular remodelling that perpetuates HF [109]. Similarly, insulin resistance in T2DM results in hyperinsulinemia which increases sympathetic tone through effects on central nervous outflow, baroreceptor reflex sensitivity and alteration of noradrenaline metabolism. However, persistently increased sympathetic activity results in insulin resistance from chronic skeletal muscle beta-adrenergic receptor activation and muscle hypoperfusion from persistent alpha-adrenergic vasoconstriction within skeletal muscles [110].

Interestingly, SGLT2-inhibitors have also shown potential in modulating sympathetic activity, thereby breaking the vicious cycle of chronic sympathetic activation in patients with T2DM and HF. Large clinical trials thus far have shown no reflex increase in heart rate following BP reduction from SGLT2-inhibition; a surrogate marker for sympathetic blockade. Mechanistic studies have supported these findings by demonstrating lower plasma metanephrines [89] and reduced noradrenaline and tyrosine hydroxylase (rate-limiting enzyme in catecholamine synthesis) activity in animal models [111]. Others have also noted that ketone bodies (which are increased with SGLT2-inhibitor therapy) attenuate sympathetic tone by suppressing G protein-coupled receptor 41 (GPR41) which is widely distributed in sympathetic ganglia [112]. These effects may also be responsible for lower sudden cardiac deaths in patients on SGLT2-inhibiton due to lower arrhythmia potential.

### SGLT2 inhibitors – a new paradigm

The pathophysiology of HF involves 2 overarching themes – abnormal loading conditions on the heart (the failing pump) and dysfunctional mechanics at the cellular level (the failing cardiomyocyte) [113]. Insofar as the treatment of chronic HF is concerned, the benefit derived from current HF therapies are ‘mechanical’ due to favourable effects on ventricular loading. For the majority of patients, in spite of optimized loading conditions, HF disease progression continues unabated due to ongoing cellular dysfunction. We propose that SGLT2-inhibitors are uniquely poised to address this important but frequently overlooked and poorly understood aspect of HF.

Nevertheless, there may be a differential effect depending on where the patient is on the HF disease spectrum. In the early stages, with adequate cellular functional reserve, optimizing the molecular milieu within the cardiomyocyte with SGLT2-inhibitor therapy (i.e. improved calcium handling, efficient myocardial energetics, optimized oxygen delivery etc.) along with modest improvements in ventricular loading (i.e. diuresis and reduced BP) confers a large benefit. However, that benefit gradually wanes as cellular dysfunction, and indeed, cellular death occurs. In such advanced circumstances, improving haemodynamic loading becomes key, with the heavy lifting being done by neurohormonal modulators and SGLT2-inhibitors playing an important, but secondary role. If this hypothesis is true, then SGLT2-inhibitors will very likely be the first therapeutic agent to be beneficial in patients with HFpEF where cardiomyocyte dysfunction predominates – we will have to wait and see if this is borne out in the large clinical trials currently underway.

## Conclusions

Some have dubbed treating HF as the “last great battle in the war on cardiovascular disease” [113]. Despite great advances in treatment options, managing HF in the modern era remains an uphill battle. SGLT2-inhibitors have demonstrated great promise in the prevention and treatment of HF and CKD. As more clinical data are collected, its therapeutic potential is being realised well beyond its initial intended use as a diabetes drug. With a clearer understanding of these molecular mechanisms, we will be able to fully harness its true potential and perhaps even pave the way for a new era of molecular therapeutic agents in this fight against HF.
